# Improvements on the Common Trephine

**Published:** 1834

**Authors:** 


### VI.—Proposed improvement of the Common Trephine.

Dr. Robert Estep, of Paris, Stark county, Ohio, has sent us, for examination, a trephine, to which he has added a guard, designed to prevent (what has sometimes happened) a wound of the dura mater and brain, by a sudden plunge of one side or the whole of the circular saw, in the latter stage of the operation.

A thread for a screw is cut for three quarters of an inch, on the hollow shaft of the trephine, beginning half an inch from the points of the teeth. Two bands are screwed on this thread. The upper of these is a mere rim, and being put on first, serves as a support for the other, which is a projecting rim of the same kind, attached to the lower margin of a hollow cylinder, half an inch in length, the inside of which is grooved into a female screw. This part is to be screwed on with the rim downwards, and this rim, when it comes to rest on the cranium, resists the further progress of the instrument. By screwing these two cylindrical bands up and down the shaft of the saw, it may be extended or limited to any depth. As they do not, in any way, embarrass the movements of the trephine, and appear to be well calculated to prevent an injury of the soft parts, towards the close of the operation, we commend them to the notice of the profession. Indeed, they seem to us both simple and ingenious; and, should experience demonstrate their utility, we would propose, that the instrument be called "Estep's Guarded Trephine."

